# Sphingolipids: the nexus between Gaucher disease and insulin resistance

**DOI:** 10.1186/1476-511X-9-113

**Published:** 2010-10-11

**Authors:** Maria Fuller

**Affiliations:** 1 Lysosomal Diseases Research Unit, Genetics and Molecular Pathology, SA Pathology [at Women's and Children's Hospital], North Adelaide, 5006, South Australia, Australia; 2 Department of Paediatrics, University of Adelaide, 5005, South Australia, Australia

## Abstract

Sphingolipids constitute a diverse array of lipids in which fatty acids are linked through amide bonds to a long-chain base, and, structurally, they form the building blocks of eukaryotic membranes. Ceramide is the simplest and serves as a precursor for the synthesis of the three main types of complex sphingolipids; sphingomyelins, glycosphingolipids and gangliosides. Sphingolipids are no longer considered mere structural spectators, but bioactive molecules with functions beyond providing a mechanically stable and chemically resistant barrier to a diverse array of cellular processes. Although sphingolipids form a somewhat minor component of the total cellular lipid pool, their accumulation in certain cells forms the basis of many diseases. Human diseases caused by alterations in the metabolism of sphingolipids are conventionally inborn errors of degradation, the most common being Gaucher disease, in which the catabolism of glucosylceramide is defective and accumulates. Insulin resistance has been reported in patients with Gaucher disease and this article presents evidence that this is due to perturbations in the metabolism of sphingolipids. Ceramide and the more complex sphingolipids, the gangliosides, are constituents of specialised membrane microdomains termed lipid rafts. Lipid rafts play a role in facilitating and regulating lipid and protein interactions in cells, and their unique lipid composition enables them to carry out this role. The lipid composition of rafts is altered in cell models of Gaucher disease which may be responsible for impaired lipid and protein sorting observed in this disorder, and consequently pathology. Lipid rafts are also necessary for correct insulin signalling, and a perturbed lipid raft composition may impair insulin signalling. Unravelling common nodes of interaction between insulin resistance and Gaucher disease may lead to a better understanding of the biochemical mechanisms behind pathology.

## Introduction

**Figure 1:**
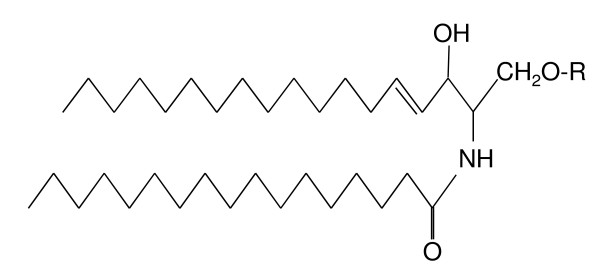
**General chemical structure of sphingolipids**. The long chain sphingosine base, generally of 18 carbons, is linked through an amide bond to a fatty acid. R = H is ceramide, the simplest sphingolipid; R = phosphocholine is sphingomyelin and R = sugars produces the glycosphingolipids including gangliosides.

Sphingolipids have become the subject of a number of biochemical processes and as such have earned the topical label of "bioactivity" [1]. Sphingolipids are also implemented in a number of disease states not only when their metabolism is affected, but when seemingly unrelated cellular homeostatic mechanisms are imbalanced. Sphingolipids are amphipathic molecules with varying degrees of hydrophobic and hydrophilic properties. The hydrophobic region comprises a long-chain base, sometimes referred to as a sphingoid base, which is linked through an amide bond to a fatty acid (Figure 1). The sphingoid base is usually 18 carbons in length, with the C_20 _being somewhat less common (reviewed in [2]). The hydrophilic region in the simplest sphingolipids can consist of just hydroxyl groups, whereas the more complex sphingolipids have phosphates and sugar residues attached. Given there are at least five different sphingoid bases present in mammalian cells with more than 20 arrangements of fatty acids differing in length of the alkyl chain and level of both saturation and hydroxylation, coupled with more than 500 carbohydrate structures reported in the glycosphingolipids, the number of possible structures is considerable [1,3]. Although a paradigm of combinatorial biosynthesis has been described to address the high degree of complexity [4], how cells deal with such lipid complexity is a central biochemical question. This can be taken to an extra dimension when considering the aberrations that can occur in a cell's management of lipids and how this is linked to disease.

Human diseases caused by alterations in the metabolism of sphingolipids are generally disorders of their degradation [5]. Traditionally the hallmark is the sphingolipidoses, named after the lipids that accumulate. They are a group of relatively rare inborn errors of metabolism caused by gene defects encoding proteins in the lysosomal degradation of sphingolipids [6]. Consequently the sphingolipid substrate for the defective protein accumulates in affected cells. Considered the prototype, Gaucher disease results from a deficiency of acid *β*-glucosidase, the enzyme responsible for the lysosomal hydrolysis of the sphingolipid, glucosylceramide, to glucose and ceramide [7]. Aside from the accumulation of glucosylceramide, a number of other secondarily stored sphingolipids reside in Gaucher disease [8,9]. A growing body of evidence now supports a role for aberrant accumulation of sphingolipids in conditions of insulin resistance [10], and this review explores the connection between sphingolipid alterations in Gaucher disease and how this is similar in insulin resistance.

## Structure of Sphingolipids

The simplest of all the sphingolipids is ceramide, which consists of a long-chain (sphingoid) base, commonly 18 carbons in length, linked to a fatty acid *via *an amide bond. The fatty acids in ceramide vary between 2 and 28 carbon atoms in the acyl chain and saturation. Ceramide provides the platform for both the synthesis and catabolism of the complex sphingolipids and is therefore often referred to as the 'hub' of sphingolipid metabolism [11]. There are three main types of sphingolipids which differ in their hydrophilic attachments onto ceramide. The first of these are the sphingomyelins which have a phosphorycholine or phosphoroethanolamine molecule with an ether linkage to the 1-hydroxy group of a ceramide; the glycosphingolipids which are ceramides with one or more sugar residues joined through a *β*-glycosidic linkage; and lastly the gangliosides which have at least three sugars, one of which must be sialic acid (Figure 1).

## Sphingolipid Metabolism

Sphingolipid metabolism is a complex interconnected network that is regulated by the synthesis and degradation of the sphingolipids themselves. Alterations in the production of individual sphingolipids effects the levels of sphingolipids that serve as substrates as well as the sphingolipid products that ensue [12]. For example, an enzyme that requires ceramide as a substrate to synthesise sphingomyelin, potentially also functions to regulate ceramide levels, as well as the many metabolites of ceramide, including sphingomyelin.

### Synthesis

*De novo *synthesis of ceramide begins with the formation of dihydrosphingosine *via *the linkage of serine with palmitic acid derived from palmitoyl-CoA. This condensation reaction is catalysed by serine palmitoyltransferase and takes place at the cytosolic leaflet of the ER [13]. The rate of *de novo *ceramide synthesis is regulated by the availability of the precursors, palmitoyl-CoA and serine [14]. Extracellular cytokines increase ceramide synthesis by up-regulating the expression of serine palmitoyltransferase [15]. Dihydrosphingosine can then undergo N-acylation with various fatty acids ranging in length from 14 to 28 carbons, with differing degrees of saturation to produce dihydroceramide, the reaction catalysed by dihydroceramide synthase. Dihydroceramide desaturase can subsequently reduce dihydroceramide to ceramide [16,17].

**Figure 2:**
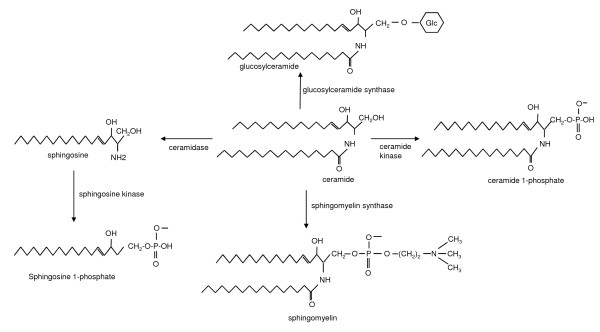
**The fate of ceramide**. Phosphorylation of ceramide by ceramide kinase generates ceramide 1-phosphate. Ceramide can be cleaved by ceramidase to produce sphingosine from which sphingosine kinase may act upon forming sphingosine 1-phosphate. Sphingomyelin is produced by the addition of phosphorycholine to the 1-hydroxyl on ceramide. Ceramide also undergoes glucosylation, producing glucosylceramide, *en route *to the synthesis of the glycosphingolipids including the gangliosides.

Once formed, ceramide becomes the precursor for the synthesis of further sphingolipid types (Figure 2). Firstly, it may undergo a simple cleavage reaction catalysed by ceramidase to produce sphingosine, which in turn serves as a substrate for sphingosine kinase to result in the formation of sphingosine 1-phosphate, a highly bioactive lipid [18]. Another recently identified bioactive lipid can be formed by phosphorylation of ceramide at the 1-position by ceramide kinase to generate ceramide 1-phosphate [19].

**Figure 3:**
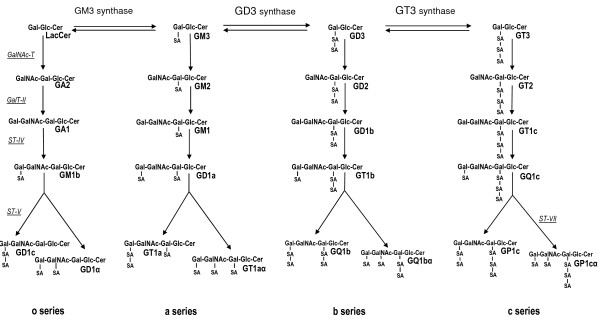
**Schematic of ganglioside synthesis**. Structures and synthetic pathways for the gangliosides. Synthesis is depicted starting with lactosylceramide (LacCer). GalNAc-T, GA2/GM2/GD2/GT2 synthase; GalT-II, GA1/GM1/GD1b/GT1c synthase; ST-IV, GM1b/GD1a/GT1b/GQ1c synthase; ST-V, GD1c/GT1a/GQ1b/GT3 synthase; ST-VII, GD1a/GT1aα/GQ1bα/GP1cα synthase.

Aside from the formation of sphingosine, sphingosine 1-phosphate and ceramide 1-phosphate, ceramide leads to the synthesis of sphingomyelin and the glycosphingolipids, including the gangliosides. Sphingomyelin is produced by the transfer of phosphorycholine from the phospholipid, phosphatidylcholine, to the 1-hydroxyl group of ceramide [20]. The first step in glycosphingolipid synthesis is the glucosylation of ceramide forming glucosylceramide (Figure 2) by the action of glucosylceramide synthase, which transfers glucose from UDP-glucose to ceramide [21]. Glucosylceramide is then converted to lactosylceramide by *β*- (1,4) transfer of galactose from UDP-galactose by galactosyltransferase I [22]. Lactosylceramide can then serve as the substrate for the synthesis of more complex sphingolipids, known as the gangliosides (Figure 3). The only known exception is G_M4_, a major component of myelin where three highly specific sialyltransferases (I, II and III) are responsible for the stepwise conversion of lactosylceramide to the mono-, di- and trisialo-gangliosides (G_M3_, G_D3 _and G_T3_, respectively) [23]. Together with lactosylceramide, these three gangliosides are precursors for the synthesis of the *o*-, a-, *b*- and *c*-series with none, one, two or three sialic residues attached to the 3-position of the galactose residue (Figure 3). The gangliosides of both the *o*- and *c*-series are only present in trace amounts in human tissue [24].

### Catabolism

Lysosomes in eukaryotic cells are the primary place for the constitutive degradation of the complex sphingolipids [25]. The stepwise breakdown of complex sphingolipids terminates with ceramide which then leaves the lysosome. Throughout different subcellular localisations, ceramide is subsequently degraded to sphingosine and a fatty acid by the action of a family of ceramidases [11].

For lysosomal degradation, complex sphingolipids must reach the endosomal/lysosomal network. A number of cellular processes assist this including endocytosis, phagocytosis, autophagy and direct transport, with the choice dependent on the source of sphingolipid [26]. Once in the lysosome, monosaccharides are sequentially cleaved by water soluble exohydrolases acting at the non-reducing end sugar in the glycosphingolipid. Glycosphingolipids are embedded in the intralysosomal membrane making the presence of sphingolipid activator proteins (SAPs) required for their degradation. SAPs function to either mediate the interaction between the membrane bound glycosphingolipid substrate and the water soluble exohydrolase or activate the enzyme directly [27]. The SAPs are five small non-enzymatic glycoproteins that are encoded by two genes. One gene encodes for the precursor of the G_M2 _activator protein and the second gene codes for prosaposin, subsequently proteolytically processed to the four SAPs A-D [28,29].

Ganglioside degradation begins with the action of the lysosomal sialidase on the multi-sialogangliosides to produce the corresponding mono-sialogangliosides, G_M1 _and G_M2_. G_M1 _is hydrolysed to G_M2 _by the removal of galactose by a *β*-galactosidase in the presence of SAP-B. *β-N*-acetyl-hexosaminidase removes *N*-acetyl-galactosamine from G_M2 _to produce G_M3_. With the aid of SAP-B, a sialidase then breaks down G_M3 _into lactosylceramide and sialic acid [30]. Subsequently, by either galactosylceramide-*β*-galactosidase or G_M1_-*β*-galactosidase a galactose is removed to produce glucosylceramide, which is then reduced to ceramide and glucose with *β*-glucosidase. Ceramide is also the product of sphingomyelin hydrolysis *via *the action of acid sphingomyelinase. Activator proteins SAP-B or -C are again required for these reactions [31,32]. Further, a phosphorycholine moiety, also a product of sphingomyelin hydrolysis, leaves the lysosome and is re-utilised. Lysosomal degradation of ceramide by acid ceramidase in the presence of SAP-D [33] is the final step and produces sphingosine and a fatty acid, which, along with other cleavage products leave the lysosome. In the cytosol, sphingosine can be phosphorylated to sphingosine 1-phosphate or can be re-acetylated back to ceramide. The non-glycosylated sphingolipids, ceramide and sphingomyelin, can also be degraded outside the lysosome in other sub-cellular locations without the need for SAPs [34]. For example, at the plasma membrane and in the Golgi, sphingomyelin can be degraded by two different sphingomyelinases to ceramide, which is subsequently cleaved by ceramidases to sphingosine which is able to be converted by sphingosine kinase to sphingosine 1-phosphate. The biological functions of ceramide and sphingomyelin, as well as their signaling metabolites, are believed to be dictated by their sub-cellular location, and that enzymes of sphingolipid metabolism regulate their cellular levels [35,36].

## Functions of Sphingolipids

Sphingolipids play a prominent role in cell signaling, acting as both first and second messengers in a variety of signaling and regulatory pathways [37]. Of all the sphingolipids, ceramide and sphingosine, together with their phosphorylated counterparts, have received the most attention with regard to bioactivity. Ceramide has been the benchmark, known for over 15 years to be involved in apoptosis [38] and cell senescence [39], and is now known to mediate many cell-stress responses, making it difficult to find a cellular process that does not involve ceramide, at least to some extent [11,36]. By modulation of signaling pathways, including pleiotropic effects on protein kinases, sphingosine has a role in inducing cell cycle arrest and apoptosis, regulating the actin cytoskeleton and endocytosis [40].

Sphingosine 1-phosphate is often referred to as the ceramide antagonist, as it is known for its cell proliferating ability by regulating cell growth, survival and proliferation, as well as cell migration and inflammation [41]. Ceramide 1-phosphate has a role in inflammation and vesicular trafficking [42], glucosylceramide in drug resistance [43] and more recently a role for dihydroceramide in cell regulation has been reported [44]. It is inevitable that as the field of lipid metabolism continues to mature, further sphingolipids and functions will be identified with 'bioactive' status. Although some mechanisms of sphingolipid action are known, (for example the pro-apoptotic function of ceramide involves binding to protein kinase Cζ [45]), efforts to determine how bioactive sphingolipids transmit their respective signals will intensify.

## Sphingolipids in Membranes

**Figure 4:**
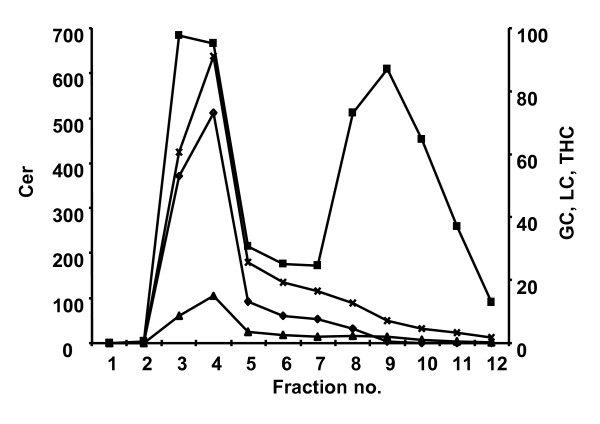
**Sphingolipid composition of lipid rafts and soluble domains in cultured skin fibroblasts**. Lipid rafts were isolated from cultured skin fibroblasts (6 mg of total cell protein) and the lipids present in each of the 12 fractions were extracted and analysed by mass spectrometry as described previously [50]. Individual species of ceramide (Cer, closed squares), glucosylceramide (GC, open diamonds), lactosylceramide (LC, closed triangles) and trihexosylceramide (THC, crosses) were summed and shown. Results are expressed as pmol lipid per mg of total protein. Lipid rafts are localised to fractions 3 and 4.

Sphingolipids are also important constituents of membranes, and together with cholesterol and the glycerophospholipids, they form the platform of eukaryotic membranes. On the surface of mammalian cells sphingolipids form patterns that are characteristic of the cell type, and alter in response to cell growth, differentiation, oncogenesis and external stimuli [46]. The lipid composition also gives rise to the different phases in the membrane, and thus begins the notion of the lipid raft hypothesis. Sphingomyelin associates with cholesterol in sphingolipid-/cholesterol-enriched domains, forming microdomains termed lipid rafts [47,48]. The fatty acid side chains of the phospholipids present in lipid rafts tend to be more highly saturated than those in the surrounding membrane. This facilitates tight packing with the saturated acyl chains of the sphingolipids and, due to the presence of cholesterol, a liquid ordered microdomain is formed that exhibits less fluidity than the surrounding membrane. Lipid rafts can be isolated by their resistance to solubilisation in non-ionic detergents and buoyancy in sucrose, and we have used this approach to characterise the lipid composition of rafts from cultured cells [49]. Figure 4 shows an example of the distribution of sphingolipids in cultured skin fibroblasts demonstrating that they predominate in the lipid raft domains rather than the soluble membrane domains. Cholesterol is believed to act as a spacer between the hydrocarbon chains of the sphingolipids holding the rafts together and, because it has a higher affinity to raft sphingolipids than to unsaturated phospholipids, it partitions between the raft and the rest of the membrane [50]. Although as much as half of the plasma membrane may be composed of rafts, it must be noted that many aspects of raft structure remain controversial, primarily because of the technical difficulties involved in their isolation and characterisation [51].

The importance of sphingolipids in cellular events has been underscored by the role of lipid rafts in intracellular signaling and vesicular trafficking [52]. Lipid rafts facilitate interactions among the lipid and protein components of signaling pathways, thereby regulating these processes. Rafts have been implicated in promoting clustering of receptors, which has been suggested to assist in the organisation of signaling molecules. It is the unique lipid composition of rafts that is believed to allow them to carry out their signaling role. What happens to signaling when the lipid composition of lipid rafts is altered is one of the most intriguing questions in lipid membrane biochemistry [53].

A seminal finding of lipid raft signaling was the observation that lipid rafts are crucial for the phosphoinositide-3 kinase (PI(3)K)/Akt signaling pathway [54], which is essential for cell physiology. Upon growth factor stimulation, PI(3)K catalyses the conversion of phosphatidylinositol (4,5)-bisphosphate to the second messenger phosphatidylinositol (3,4.5)-triphosphate, that recruits Akt from the cytosol to the plasma membrane. Here, Akt goes through two phosphorylation events, one at a threonine residue at the T-loop of Akt [55] and another at a serine residue located at the C-terminal region of the molecule [56], catalsyed by phosphoinositide-dependent kinase 1 and 2, respectively, to activate Akt. Both the phosphoinositide-dependent kinases and Akt are located in lipid rafts and disturbing raft lipid composition alters cell survival and metabolism *via *the PI(3)K/Akt signaling pathway [57,58].

## Inborn Errors of Sphingolipid Metabolism

The importance of efficient sphingolipid degradation is illustrated by the existence of inherited lysosomal glycosidase and activator protein deficiencies that lead to a group of lysosomal storage disorders known as the sphingolipidoses [5]. The sphingolipidoses consist of at least nine disorders, each resulting in the accumulation of sphingolipids within the lysosomes of affected cells (Table 1). The primary substrate that accumulates in the lysosome, the substrate for the enzyme deficiency, leads to a range of pathology that can involve the liver, spleen, kidney, bone and central nervous system. Although the primary enzymatic defects and the resulting clinical pathologies have been well characterised for these disorders, there is little known about the biochemical processes leading to clinical pathology.

**Table 1:** The sphingolipidoses

disease	clinical phenotype	OMIM number	enzyme deficiency	primary stored sphingolipids
G_M1_-gangliosidosis types I/II/III		230500	*β*-galactosidase	G_M1 _and G_A1 _ganglioside

G_M2_-gangliosidosis type A/B		272750	G_M2_-activator deficiency	G_M2 _ganglioside

G_M2_-gangliosidosis type I (B variant)	Tay-Sachs disease	272800	*β*-hexosaminidase A	G_M2 _ganglioside

G_M2_-gangliosidosis type II (O variant)	Sandhoff disease	268800	*β*-hexosaminidase A and B	G_M2 _and G_A2 _ganglioside, and globoside

Gaucher disease		230800 176801	acid *β*-glucosidase or SAPC or LIMP-2	glucosylceramide

Fabry disease		301500	α-galactosidase A	ceramide trihexoside

metachromatic leukodystrophy		250100 249900	arylsulphatase A or SAPB	sulphated galactosylceramide

globoid cell leukodystrophy	Krabbe disease	245200	*β*-galactocerebrosidase	galactosylceramide
Niemann-Pick type A/B	Niemann-Pick disease	257200	acid sphingomyelinase	sphingomyelin

Farber lipogranulomatosis	Farber disaese	301500	acid ceramidase	ceramide

OMIM, Online Mendelian Inheritance in Man

A fundamental question is how the lysosome accumulates sphingolipids that can differ in as little as two hexose attachments, can display such a disparate phenotype. For example, in Fabry disease, where the lysosomal accumulation of ceramide trihexoside manifests as acroparesthesias, angiokeratoma and occlusive vascular disease of the kidney, heart or brain, compared with Gaucher disease where accumulation of a monohexosylceramide leads to massive hepatomegaly and splenomegaly. Such diverse and extensive array of clinical manifestations suggests that many secondary biochemical pathways involving, but not limited to, sphingolipids must also be affected [59]. Beside the accumulation of the primary sphingolipid substrate in the sphingolipidoses, secondary sphingolipid alterations have also been reported [8,60,61]. Perhaps this is not surprising given the homeostatic response that would come into play in attempt to restore the sphingolipid balance when the degradation of one is impaired. It has been hypothesised that an accumulation of lipids in lipid rafts in late endosomes/lysosomes has a role in the pathogenesis of the sphingolipidoses [62]. This is evidenced in Niemann-Pick C disease where cholesterol/sphingolipid accumulation causes an overcrowding of rafts in the endocytic network to form multilamellar bodies [63]. Additionally, alterations in lipid raft composition occur in Gaucher disease [49].

### Gaucher Disease

Gaucher disease is often considered the prototype for the sphingolipidoses; not only is it the most prevalent lysosomal storage disorder [64] it was also the first to be described [65]. As such it has served as a model for the treatment of other lysosomal storage disorders and inborn errors of metabolism [66,67]. Gaucher disease is inherited in an autosomal recessive manner and results from the deficiency of acid *β*-glucosidase, the enzyme responsible for the lysosomal hydrolysis of the sphingolipid, glucosylceramide, to glucose and ceramide. Insufficient acid *β*-glucosidase activity from more than 300 mutations in its gene has been shown to affect its catalytic function, intracellular stability and/or trafficking [68,69]. This enzyme dysfunction leads to the lysosomal accumulation of glucosylceramide, which is also a metabolic intermediate derived from the cellular turnover of membrane gangliosides and globosides. The major site of glucosylceramide storage is the cells of the mononuclear phagocyte system, especially those in the liver, spleen, lung and bone marrow. The accumulation of excess glucosylceramide in macrophages is the main manifestation in visceral organs, leading to hepatosplenomegaly, anaemia and thrombocytopaenia and bone involvement. Less common is lung involvement, as well as many other rather inconsistent manifestations [7]. Although the pathology of Gaucher disease primarily results from the storage of sphingolipids in tissues throughout the mononuclear phagocyte system with subsequent macrophage activation and tissue inflammation, the mechanisms behind disease remain ill-defined [70].

The disease has been broadly categorised into three clinical subtypes based on the presence and the rate of rapidly advancing central nervous system pathology: type 1 is the non-neuronopathic form exhibiting only the visceral manifestations, and types 2 and 3 are the acute and sub-acute neuronopathic variants, respectively. Type 2 is generally seen in infancy with a rapid neurodegenerative course and death usually within the second year of life. Type 3 generally progresses with a more chronic course, a later onset and a slower neurodegenerative path than type 2. Type 1 is more common, with an estimated incidence of about 1 in 855 live-births in the Ashkenazi Jewish population [7] and age of onset highly variable. As with all of the sphingolipidoses, a broad spectrum of phenotypes exists as do some general genotype/phenotype correlations [71]. The advent of enzyme replacement therapy has revolutionised the outlook for individuals with type 1 Gaucher disease [67]. This therapy is very effective in reducing organ size and correcting haematological abnormalities, however its effect on skeletal and other complications of disease is not so straightforward [72].

### Sphingolipid Related Cellular Pathobiology

Although the characteristic sphingolipid laden macrophages are the hallmark of Gaucher disease, resulting in macrophage activation and tissue inflammation, it is clear that other homeostatic alterations underlie disease symptomatology. With respect to sphingolipids, the primary storage of glucoslyceramide leads to secondary alterations of sphingolipids that have been reported to affect gangliosides in various tissues [73] and sphingomyelin in cell models of Gaucher disease [74]. We have also shown secondary increases in ceramide, di- and trihexosylceramides in a macrophage model of the disorder [8] and in fibroblasts from Gaucher patients [9]. Moreover, this secondary sphingolipid accumulation, as well as glucosylceramide, was shown to extend beyond the lysosome implying interference with biochemical pathways at extralysosomal sites. It has also been proposed that the secondary accumulation of other sphingolipids such as ceramide, sphingosine and sphingosine 1-phosphate act as signaling intermediaries to produce an activation of macrophages, with the subsequent release of pro-inflammatory cytokines [75].

The skeletal complications of Gaucher disease likely relate to humoral factors produced by lipid laden macrophages that ultimately alter bone remodelling [76]. The observation of the critical role of circulating sphingosine 1-phosphate in bone homeostasis suggests involvement of this sphingolipid in Gaucher disease [77]. In fact reduced sphingosine 1-phosphate levels have been observed in mesenchymal stromal cells with chemically induced inhibition of *β*-glucosidase to mimic the Gaucher disease phenotype [78]. Additionally in this model, and in mesenchymal stromal cells from a type 1 Gaucher patient a number of inflammatory mediators were found to be upregulated. As sphingolipids are involved in inflammation and apoptosis, they may have a direct activating or enhancing effect on macrophage function, which has been earlier postulated to be mediated through calcium channel dysfunction [79]. Glucosylceramide and its catabolism to ceramide is, at least in part, believed to have an immunomodulatory effect by enhancing dendritic cells as well as natural killer and regulatory T cells [80]. Although many of these processes underlie these secondary disturbances, much still remains to be learned to delineate the metabolic pathways involved and the "triggers" for these biochemical processes.

Recently the catabolic processes that occur in the lysosome were demonstrated to be directly involved in lipid metabolism [81]. The key finding was that the process of autophagy regulates lipid metabolism, by mobilising intracellular lipid stores as an additional source of energy. This indicates that lysosomes do not fuse directly with cholesterol and triglyceride contained in stored lipid droplets but rather fuse with lipid droplet containing phagosomes. Although the exact mechanisms of degradation of lipid droplets through autophagy remain to be elucidated, it provides evidence for a role for autophagy and lysosomal degradation in lipid metabolism. [82]

## The effect of Sphingolipids on Insulin Sensitivity

Insulin resistance is defined as the reduced ability of a cell to respond to physiological concentrations of insulin. In normal physiological conditions following insulin stimulation, insulin binds to the insulin receptor and induces autophosphorylation *via *the receptor's intrinsic tyrosine kinase. The activated receptor phosphorylates a family of insulin receptor substrate proteins which initiates at least two signaling cascades. Firstly, Akt together with protein kinase C, promotes translocation of the glucose transporter (GLUT-4) to the plasma membrane enabling the uptake of glucose, and secondly, the mitogen activated protein kinase cascade is initiated. Interestingly, this latter cascade is not involved in either insulin stimulated glucose transport or glycogen metabolism [83]. The PI(3)K/Akt signaling pathway however, is central to proper insulin signaling [84].

As discussed above, lipid rafts are required for effective PI(3)K/Akt signaling. Evidence suggests that as there is a requirement for the spatiotemporal organization of signaling components in plasma membrane domains for effective insulin signaling [85]. Coupled with the finding of the insulin receptor in lipid rafts [86], the nature of the sphingolipids in these domains may be one of the determining factors in the functioning of these signaling components and thereby insulin signaling. A number of studies would support this and imply that one of the many causes of insulin resistance is related to altered sphingolipid metabolism. Firstly, ceramide is known to inhibit insulin signaling *via *the Akt signaling pathway, effectively blocking Akt activation. The mechanism prevents the translocation of Akt to the plasma membrane and activation of the phosphoinositide-dependent kinase 2, which impairs Akt activation by removing the activating phosphates [87]. Increased concentrations of ceramide have been reported in skeletal muscle biopsies from obese humans with insulin resistance [88], but further studies have yielded conflicting results. Skovbro et al. [89] reported that ceramide failed to increase in both insulin resistant and type 2 diabetic patients compared to individuals with a normal insulin response. Additionally, work reported by Holland et al. [90] suggest that the types of oils (lard oil high in saturated fatty acids, versus soy bean oil, primarily unsaturated fatty acids) used in these experiments may explain some of the disparity, as unsaturated fat has been shown to cause insulin resistance independently of ceramide. Ceramide debate is likely to continue until a consensus on its contribution to insulin resistance can be achieved [91].

G_M3 _is another sphingolipid believed to interfere with insulin signaling. Experiments *in vivo *using G_M3 _synthase knockout mice showed enhanced tyrosine phosphorylation of the skeletal muscle insulin receptor when compared with wild-type mice [92]. The addition of G_M3 _to cultured 3T3-adipocytes was shown to suppress insulin stimulated tyrosine phosphorylation of the insulin receptor and its downstream substrate, resulting in impaired glucose uptake [93]. A proposed mechanism for this is that G_M3 _displaces the insulin receptor in lipid rafts, impairing insulin receptor interaction and effectively decreasing insulin receptor dependent signaling [94]. In the lipid rafts the amount of G_M3 _doubled whereas it remained unchanged in untreated 3T3-adipocytes. Nonetheless the integrity of the insulin receptor in rafts may be maintained *via *interaction with lipid raft proteins such as caveolin. The potential role of other sphingolipids in insulin resistance is somewhat restricted. Sphingosine has received some attention, as has glucosylceramide, although mechanisms of action are purely speculative at this stage [95]. Further research to link these mechanisms to impaired lipid raft function due to interactions between sphingolipids present in the rafts and the insulin receptor, as well as Akt and other potential signalling pathways is needed.

## Insulin Resistance and Gaucher Disease

The connection of sphingolipids to insulin resistance has emanated from experimental findings outlined above, demonstrating that sphingolipid metabolism influences insulin sensitivity. It follows that Gaucher disease, a genetic disorder of sphingolipid metabolism, is a useful human model to investigate the potential role of sphingolipids in insulin resistance. To this end, impaired insulin mediated glucose uptake has been demonstrated in patients with Gaucher disease compared with unaffected controls [96]. Although the number of Gaucher patients investigated was only six making it difficult to determine how widespread insulin resistance might be in the Gaucher population. A subsequent study that included larger numbers of Gaucher patients, some of whom were receiving enzyme replacement, showed that there was no increase in the prevalence of type II diabetes in Gaucher patients [97]. Interestingly, this study found that the number of these Gaucher patients that were overweight was less than in the general population, and further findings of Ucar et al. [98] reported that insulin resistance in Gaucher disease was not related to overweight.

Energy balance is altered in mice models of lysosomal storage disorders. Both a deficiency in adipose storage and lower leptin levels were demonstrated in five different lysosomal storage disorders, highlighting the involvement of lipid metabolism [99]. This is in support of earlier findings reporting increased energy expenditure in Gaucher patients [100,101]. Clearly metabolic abnormalities are apparent in Gaucher disease but the administration of replacement enzyme to reduce glucosylceramide levels in patients seems to bring about its own metabolic consequences such as peripheral insulin resistance [97]. It is not clear whether this is due to reductions in glucosylceramide levels *per se *or secondary sphingolipid alterations, such as transient increases in ceramide as the excess glucosylceramide passes through the catabolic pathways. Recently it has been suggested that individuals with Gaucher disease have a selective advantage against some systemic disorders due to the immunomodulatory effects of glucosylceramide promoting dendritic cells, natural killer and regulatory T cells [81]. This must be related at least in part to restricted catabolism of glucosylceramide to ceramide.

**Figure 5:**
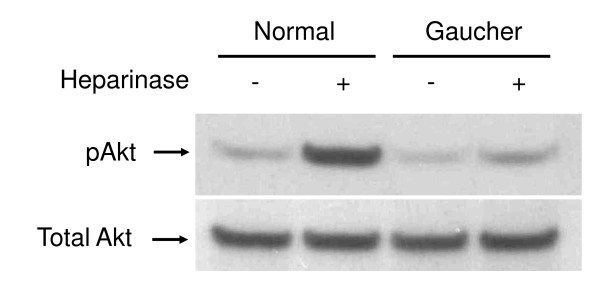
**Western blot analysis of Akt phosphorylation in a Gaucher cell model**. A Gaucher cell phenotype was induced in THP-1 macrophages with conduritol B epoxide as described previously [8]. Cells were stimulated for 5 minutes with 1 μg/ml heparinase (Sigma), extracts prepared and probed for the phosphorylated form of Akt (Ser473) (top panel) and total Akt (bottom panel). Antibodies were purchased from Cell Signaling Technology.

Aside from the primary storage material, glucosylceramide, a number of other lipids are also altered in Gaucher disease. For example, we have observed elevations in G_M3 _in Gaucher disease [102], and elevations in G_M3 _have been posited as playing a role in the insulin resistance apparent in Gaucher disease [103]. Insulin resistance has been referred to as a lipid raft disorder, primarily on the basis of accumulation of G_M3 _which results in a loss of the insulin receptor from lipid rafts [104,105]. Lipid rafts have also been shown to be affected in Gaucher disease [50]. The altered lipid raft composition is likely to affect Akt signaling, (as discussed above), and preliminary data we have generated using a Gaucher cell model suggest a role for the PI(3)/Akt signaling pathway in Gaucher disease (unpublished). Figure 5 shows that Akt phosphorylation is impaired in a Gaucher cell model generated by chemical inhibition of enzyme activity [50]. Thus, there is a clear link implicating altered lipid raft composition and impaired Akt signaling in both Gaucher disease and insulin resistance.

Probably the most direct pathogenic feature connecting Gaucher disease with insulin resistance is macrophage activation leading to inflammation [71,106]. In mice, insulin resistance caused by diet-induced obesity produced a switch in macrophage polarisation from M2 to M1 [107]. Alternatively activated macrophages with anti-inflammatory properties are considered M2, whereas M1 refer to classically activated macrophages which secrete pro-inflammatory cytokines such as IL-6 and TNFα, both of which are elevated in Gaucher disease and insulin resistance. This begs the question whether M1 macrophages share similarities in Gaucher disease and insulin resistance. The emerging scene depicts macrophages subjected to an excessive lipid load, which, in turn determines their function, and has a substantial impact on cell, organ and tissue homeostasis. The picture in Gaucher disease is one of enlarged liver and spleen and insulin resistance reveals macrophage infiltration of adipose tissue and muscle. The term lipotoxicity has been used to describe the continued accumulation of lipids, and lipotoxicity in macrophages may be at the cross-roads of Gaucher disease and insulin resistance.

Reducing sphingolipid synthesis by inhibiting glucosylceramide synthase has been a therapeutic strategy for Gaucher disease, but also corrects insulin resistance in cultured adipocytes from obese individuals and in obese rodents [108,109]. In doing so inflammation was also shown to be mitigated as evidenced by reduced macrophage numbers and macrophage chemo attractants. Inflammation is also a key pathogenic feature in Gaucher disease highlighting common elements between the two conditions. Continuing to explore the interconnections between Gaucher disease and insulin resistance in the future is likely to identify shared pathogenic aspects which may present some novel opportunities for treatment.

## Concluding Remarks

In closing, the link between lysosomal storage disorders and insulin resistance as membrane related disorders has been known for more than 20 years, but the mechanisms are yet to be clarified. Evidence exists that these mechanisms at least in some part, involve sphingolipids through both their bioactive and structural (membrane) properties, and thus converge on the lipid raft hypothesis. On reflection, much has been learnt about the role that sphingolipids play in the biochemistry of disease, but we are a long way from knowing the exact permutations and combinations of sphingolipids involved. The manipulation of sphingolipid metabolism using drugs and siRNA to specifically alter the cell's sphingolipid composition will be an important experimental avenue to pursue. To this end Gaucher disease will be a useful human model to study the relationship between sphingolipid metabolism and insulin resistance. One of the questions that remain unanswered is whether blocking the catabolism of ceramide or other sphingolipids has any impact on insulin sensitivity? Technology is advancing rapidly and tools such as mass spectrometry now enable a detailed analysis of sphingolipids in complex biological samples [110]. Unfortunately we are still faced with the limitation of only being able to take a snapshot of a cell at one particular point in time which makes it difficult to put the pieces of the jigsaw together to unravel what is going on biochemically in "real time". Thus, we are left with the paradoxical aim of integrating both the biology and function of sphingolipids in a dynamic system, yet at a stage where only static measurements can be made. No doubt animal models will be used to simulate the framework of interconnected events in the immediate term, until this shortcoming can be overcome.

## Competing interests

The author declares that they have no competing interests.

## Author Details

Lysosomal Diseases Research Unit, Genetics and Molecular Pathology, SA Pathology [at Women's and Children's Hospital], 72 King William Road, North Adelaide, South Australia, 5006, Australia
